# Cold Feet

**Published:** 1873-04

**Authors:** 


### COLD FEET.

Persons who have cold feet habitually, should remember that it is a symptom of disease always; a symptom which arises from want of general good health, which in turn is owing to what we call dyspeptia, which means that the stomach has not the power to work up the food in a way to get all the nourishment out of it, so that it can be converted into healthy blood. Unhealthy blood is deficient in warmth; hence, it does not warm the body; there never was a dyspeptic who was not chilly; he gets cold easily; he must bundle up whenever he goes out of doors; and if the body is cold, much more will the feet and hands be cold. Hence, the most effective method of getting rid of cold feet is to secure a higher state of general health by eating regularly at not less than five hours interval and spending a large portion of the time in out-door activities. Yet a good deal may be done towards keeping the feet warm by holding them in warm water, half way up the ankles, both in at once, for five minutes, then put them in cold water, deep enough only to cover the toes, long enough to count sixty, then rub the soles pretty hard and briskly with a coarse cloth; next hold them to the fire, rubbing the soles with the hand, until most perfectly dry, draw on the stockings and take a brisk walk; do this every morning. The feet of some are kept more comfortable with cotton than woolen; others wear two pairs of thin stockings; each one must experiment on himself. White woolen stockings are warmer than colored.

